# Antimicrobial Efficacy of Garlic (*Allium sativum*) Extract Against *Aeromonas Hydrophila* Isolated From Diseased Pangasius Catfish

**DOI:** 10.1002/vms3.70724

**Published:** 2025-12-02

**Authors:** Sumaia Siddique, Md. Mubarack Hossain, Md. Nurul Haider

**Affiliations:** ^1^ Department of Fisheries Technology Bangladesh Agricultural University Mymensingh Bangladesh

**Keywords:** *Aeromonas hydrophila*, antimicrobial activity, aquaculture, garlic extract, pangasius catfish

## Abstract

**Background:**

Pangasius catfish (*Pangasianodon hypophthalmus*) is one of the most commercially and nutritionally valuable aquaculture species in Bangladesh. In recent years, outbreaks of bacterial diseases, particularly those caused by *Aeromonas hydrophila*, have posed serious challenges to its production. The frequent and often indiscriminate use of antibiotics for disease management has intensified the problem of antimicrobial resistance, emphasising the urgent need for safe, effective and sustainable alternatives.

**Objectives:**

This study assessed the antibacterial potential of garlic (*Allium sativum*) extracts against *A. hydrophila* isolated from infected Pangasius catfish.

**Methods:**

A total of 49 bacterial isolates were collected from different commercial Pangas fish farms located in Trishal, Muktagacha and Phulpur upazilas of Bangladesh. All isolates were molecularly confirmed as *A. hydrophila*. The antibacterial activity of aqueous and methanolic garlic extracts was subsequently evaluated using disc diffusion, minimum inhibitory concentration (MIC) and minimum bactericidal concentration (MBC) assays.

**Results:**

The aqueous extract exhibited no detectable antibacterial activity, whereas the methanolic extract demonstrated marked inhibitory effects against *A. hydrophila*. The largest inhibition zone (15.11 ± 0.05 mm) was observed at 8 mg/mL of garlic extract in 20% methanol. MIC values ranged between 6 and 8 mg/mL, while MBC assays confirmed bactericidal activity at 8 mg/mL in 20% methanol. These findings indicate that the methanolic extract exhibited significant antibacterial properties and could be recommended as a phytobiotic alternative to antibiotics for managing *A. hydrophila* infections in Pangasius aquaculture.

**Conclusion:**

Methanolic extracts of garlic exhibit significant antibacterial activity against *A. hydrophila* and could be recommended as a phytobiotic alternative to conventional antibiotics for managing *A. hydrophila* infections in Pangasius aquaculture. This natural approach may contribute to sustainable fish health management and mitigate the development of antimicrobial resistance.

## Graphical abstract

The study evaluated the antibacterial efficacy of garlic (*Allium sativum*) extracts against *Aeromonas hydrophila* isolated from diseased Pangasius catfish. Methanolic extract exhibited significant antibacterial activity, producing the largest inhibition zone (15.11 ± 0.05 mm) at 8 mg/mL and showing MIC and MBC values at 6–8 and 8 mg/mL, respectively. The findings highlight methanolic garlic extract as a promising natural phytobiotic alternative to antibiotics in Pangasius catfish aquaculture.

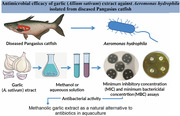

## 1 Introduction

Disease outbreak is one of the most serious constraints to sustainable aquaculture worldwide (Kowalska et al. 2020; Caputo et al. 2023). Infectious pathogens reduce productivity and cause major economic losses, with disease outbreaks accounting for nearly 50% of global production losses and an estimated annual cost of $6.5 billion (Ahmed et al. 2025). Among the various diseases affecting cultured fish, bacterial infections remain particularly problematic. Intensive aquaculture practices, poor water quality, overstocking and stress factors facilitate opportunistic bacterial infections (Kumar et al. 2025; Hossain et al. 2021; Bhuiyan et al. 2022).

Numerous bacterial genera have been reported and isolated from farmed fish, including *Streptococcus*, *Edwardsiella*, *Flavobacterium*, *Mycobacterium*, *Escherichia*, *Vibrio* and *Salmonella* (Afsana et al. 2025; Samiappan et al. 2022; Hossain et al. 2024; Bhuiyan et al. 2022). Among these, *Aeromonas hydrophila* is considered one of the most virulent, causing Motile Aeromonas Septicemia (MAS), a severe ulcerative disease in farmed and wild fish (S. T. Wang et al. 2017; Marinho‐Neto et al. 2019). Beyond fish, *Aeromonas* species can infect amphibians, reptiles and mammals, raising additional concerns for public health (Janda and Abbott 2010; Plumb and Hanson 2010).

The Pangasius catfish (*Pangasianodon hypophthalmus*), a benthopelagic and potamodromous species, has become an aquaculture mainstay due to its rapid growth, high stress tolerance and artificial propagation success (Bhujel 2024). It also represents a major export commodity for Asian countries, including Bangladesh, India, Vietnam and Thailand (Singh 2025; Hassan et al. 2025; Haque et al. 2021). Yet, intensive farming has increased the prevalence of infectious diseases in Pangasius aquaculture (Abedin et al. 2020). Most notably, *A. hydrophila* is responsible for a large proportion of losses in Pangasius farming, reaching up to 30%–50% during the grow‐out stage (Tran et al. 2025).

Antibiotics are widely applied in aquaculture for prophylaxis, therapy and growth promotion (Rigos et al. 2024; Yilmaz et al. 2024). However, antimicrobial resistance (AMR) has become a critical global issue (Y. Wang et al. 2025; WHO 2024). Resistant bacteria from aquaculture environments may transfer to terrestrial systems and humans, exacerbating the public health burden (Granados‐Chinchilla and Rodríguez 2017). Thus, reducing antibiotic dependency is a priority.

Plant‐derived bioactive compounds offer promising alternatives to conventional antibiotics. Their accessibility, low cost and biodegradability make them attractive for aquaculture applications (Gabriel et al. 2022; Haniffa and Kavitha 2012; Olusola et al. 2013). Among these, garlic (*Allium sativum*) has emerged as one of the most promising natural compounds, demonstrating strong anti‐infective activity against microorganisms. The antimicrobial effects of garlic are attributed mainly due to its sulphur‐containing molecules, such as ajoene and vinyldithiins, along with polyphenolic compounds, including rutin, quercetin, gallic acid, pyrogallol, resorcinol, protocatechuic acid and kaempferol, which play important roles in its activity. In addition, enzymes such as alliinase, peroxidase and myrosinase have broad‐spectrum antibacterial, antifungal and antiviral effects (Mosaddad et al. 2023; Bhatwalkar et al. 2021; Tariq et al. 2019). Several studies have shown that garlic extract can effectively inhibit the growth of diverse bacterial and fungal pathogens, including *Staphylococcus aureus, Streptococcus* spp.*, Escherichia coli, Shigella senteriae, Salmonella* spp.*, Klebsiella* spp.*, Proteus mirabilis and Helicobacter pylori*, as well as fungi such as *Fusarium oxysporum*, *Botrytis cinerea*, *Verticillium dahliae* and *Phytophthora capsici* (Rameshwar et al. 2021; Sikandar et al. 2020; Mougou et al. 2018).

Since Pangasius farming is significantly threatened by *A. hydrophila* infections, which cause substantial economic losses, and conventional disease control has predominantly relied on antibiotics, contributing to the emergence of AMR and raising serious environmental and public health concerns, there is a growing need to explore natural, eco‐friendly alternatives to synthetic antibiotics. Among plant‐derived compounds, garlic has demonstrated potent antimicrobial activity against a wide range of pathogens. Therefore, this study was undertaken to isolate and identify *A. hydrophila* from diseased Pangasius catfish and to evaluate the antimicrobial potential of garlic extracts against this bacterium to offer a natural alternative to antibiotics for sustainable disease management in Pangasius aquaculture.

## 2 Materials and Methods

### 2.1 Study Area

**FIGURE 1:**
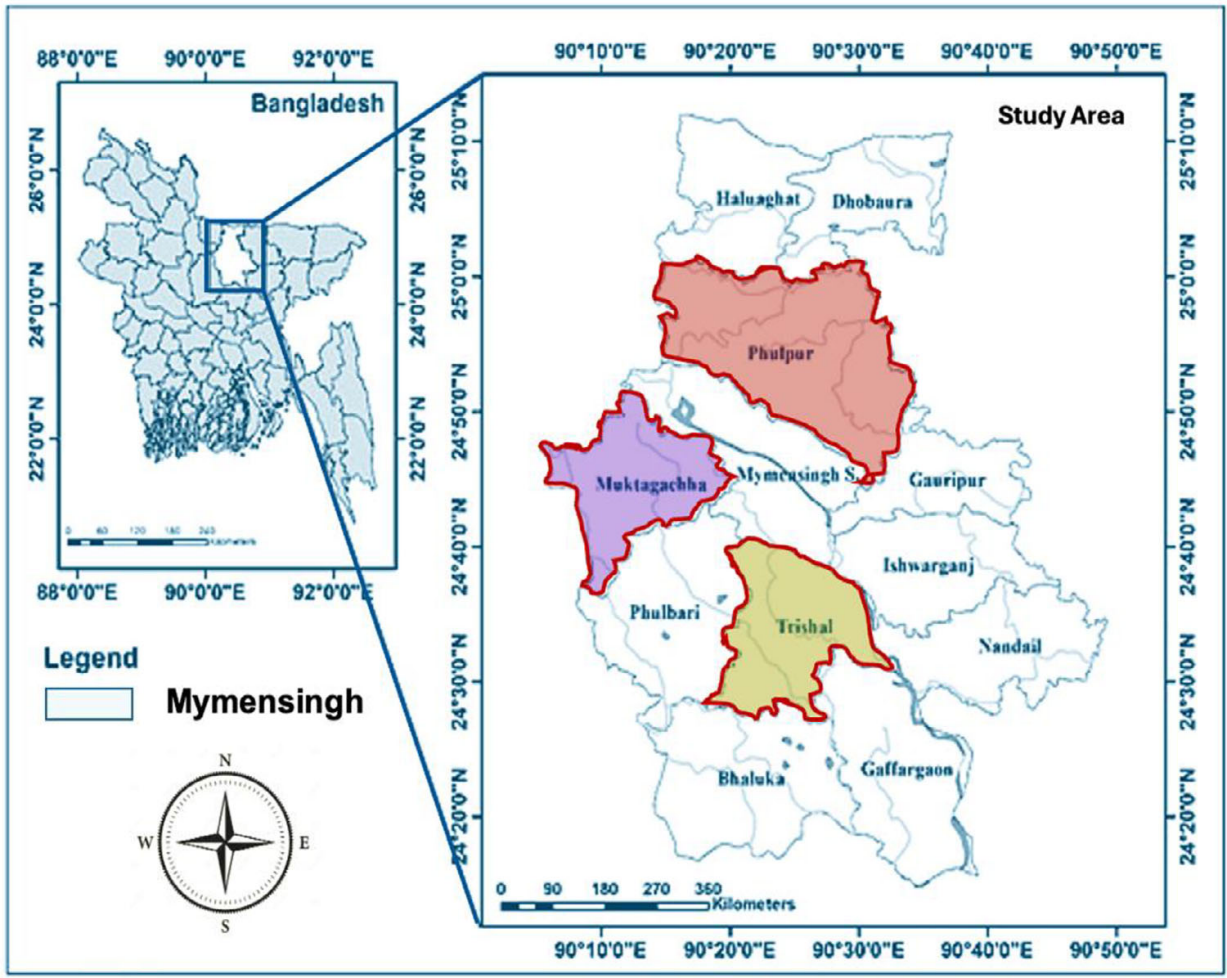
Map illustrating the location and spatial distribution of Pangasius catfish farms in the Mymensingh district, Bangladesh. The map was generated using a Geographical Information System DIVA‐GIS and visualized with ArcGIS Pro 3.5.

Commercial Pangasius aquaculture farms located in Trishal, Muktagacha and Phulpur upazilas within Mymensingh district of Bangladesh (Figure 1) were selected to collect infected Pangasius catfish samples for the isolation and identification of *A. hydrophila* and subsequent evaluation of AMR patterns.

### 2.2 Isolation of Suspected *A. hydrophila*

#### 2.2.1 Sample Collection

A total of 10 commercial Pangasius farms were selected, and moribund fish were sampled based on external clinical signs such as skin, fin and gill haemorrhages; fin rot and tail erosion; abdominal distension; exophthalmia; and skin ulcers suggestive of MAS. The fish were placed in sterile insulated containers with ice packs and transported to the Fisheries Microbiology Laboratory, Bangladesh Agricultural University, Mymensingh, Bangladesh.

#### 2.2.2 Bacteriological Examination

Collected diseased Pangasius fish were dissected under sterile conditions. To isolate bacteria, samples were aseptically collected from the kidney, liver, spleen, skin and eye lesions using sterile inoculation loops. Bacterial isolation was carried out on Trypticase Soy Agar (TSA; Becton, USA) following the method described in Ngo et al. (2022), with minor modifications. Briefly, the inoculated TSA plates were incubated at 24°C for 48 h. After incubation, distinct colonies were selected based on their morphological characteristics and subcultured onto fresh TSA plates to obtain pure 24‐h cultures. To selectively identify *Aeromonas* spp., these pure cultures were streaked onto *Aeromonas*‐selective agar. Only isolates that exhibited growth on the selective medium were considered presumptive *Aeromonas* and retained for further analysis.

### 2.3 Molecular Detection of *A. hydrophila*

#### 2.3.1 Pre‐Enrichment and DNA Extraction

The previously obtained isolates were incubated in nutrient broth at 30°C overnight, and genomic DNA was extracted through the boiling method (Zhou et al. 2007). In brief, 1 mL of culture was centrifuged at 5000 rpm for 3 min, washed twice in sterile distilled water, and resuspended in 200 µL of distilled water. The suspension was boiled for 10 min, chilled on ice, and centrifuged at 10,000 rpm for 10 min. The supernatant served as the polymerase chain reaction (PCR) template and was stored at −20°C for further use.

#### 2.3.2 PCR Amplification

**FIGURE 2:**
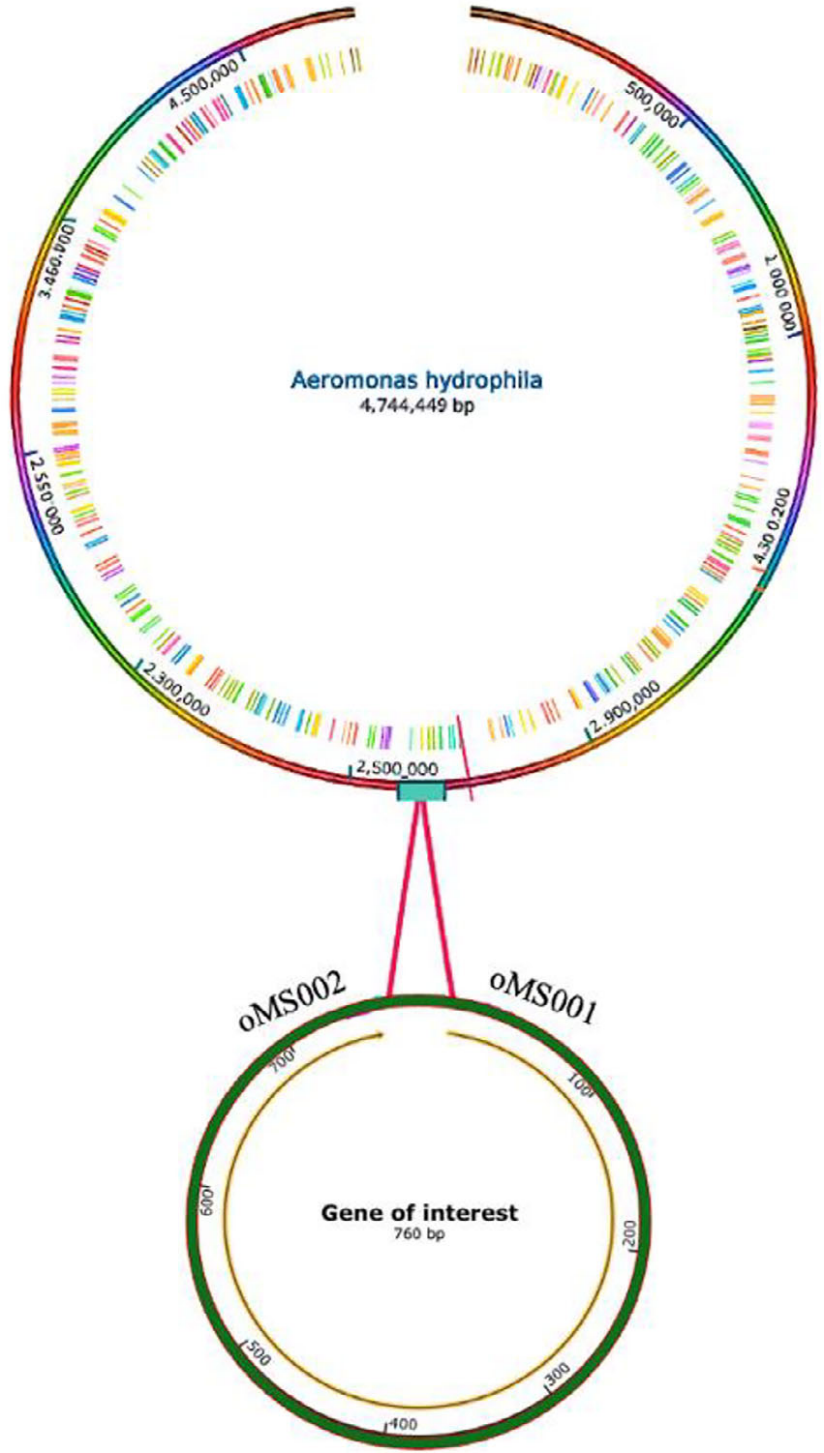
Schematic representation of the *Aeromonas hydrophila* genome highlighting the location of the *lip* gene fragment. The genome map was generated and visualized via SnapGene 6.2.

The target gene sequence of the *lip* gene of *A. hydrophila* encoding a thermostable extracellular lipase was retrieved from the National Center for Biotechnology Information (NCBI) database based on the available genome assembly NC_008570.1 (Figure 2). Specific primers were designed by using primer3 to amplify the gene of interest for molecular detection (Table 1). Amplification was performed with a DNA thermal cycler (Mastercyclear, Eppendorf, Humburg, Germany) with some modifications as follows: the reaction mixture consisted of 1 µL of Taq polymerase (1 unit), 5 µL of 10X PCR amplification buffer (100 mM Tris‐HCl, 25 mM MgCl_2_, 500 mM KCl, pH 8.3), 3 µL of deoxynucleoside triphosphate (100 µM), 0.5 µL of each primer (100 pmol) and double distilled water up to a final volume of 50 µL. A total of 40 PCR cycles were carried out under the following thermal cycling conditions: initial denaturation at 90°C for 4 min, followed by denaturation at 94°C for 60 s, annealing at 65°C for 60 s, and extension at 72°C for 90 s. A final extension step was performed at 72°C for 5 min. The PCR products were analysed by agarose gel electrophoresis using a 100 bp DNA Ladder (Promega, USA).

**TABLE 1:** List of primers used for amplification of the *lip* gene of *Aeromonas hydrophila* isolated from diseased Pangasius catfish from different farms in the Mymensingh district, Bangladesh.

Species	Name of primer	Primer sequence (5′–3′)	Amplicon size (bp)
*Aeromonas hydrophila*	oMS001	AACCTGGTTCCGCTCAAGCCGTTG	760
	oMS002	TTGCCTCGCCTCGGCCCAGCAGCT	

### 2.4 Preparation of Garlic Extracts and Determination of Antimicrobial Activity Against *A. hydrophila*

Fresh garlic was purchased from a local grocery store. After cleaning and peeling, the cloves were sliced to access the edible part. Two types of garlic extracts were prepared: aqueous and methanolic. Briefly, 10 g of garlic was crushed in a sterile mortar and pestle and homogenized with a blender. Five grams of the homogenate was separately soaked in 100 mL of distilled water (for the aqueous extract) and in 10%, 20% and 40% methanol (for the alcoholic extracts). The mixtures were subsequently centrifuged at 10,000 rpm for 5 min, after which the supernatants were collected, followed by a second centrifugation (10,000 rpm, 5 min) to remove any remaining debris. The aqueous extract served as a control. The extracts were stored at −20°C until use.

### 2.5 Antibacterial Assay

The antibacterial activity of garlic extracts against *A. hydrophila* was evaluated through the disc diffusion method (Durairaj et al. 2009). Briefly, sterile 6 mm filter paper discs (Whatman, 0.1 mm) were impregnated with 100 µL of garlic extract at concentrations of 1, 2, 4, 6, and 8 mg/mL and allowed to air dry to ensure absorption. Overnight cultures of molecularly identified *A. hydrophila* isolates were prepared in trypticase soy broth (TSB) at 30°C and swabbed onto *Aeromonas* selective agar plates. The extract‐impregnated discs were placed on the inoculated plates alongside the control discs containing only aqueous extract. After a 30‐min adsorption period, the plates were incubated at 30°C for 24 h. Antibacterial activity was assessed by measuring the diameter of the inhibition zones around the discs.

### 2.6 Determination of Minimum Inhibitory and Minimum Bactericidal Concentrations

Minimum inhibitory concentration (MIC) of garlic extracts was determined via the broth dilution method (Fani et al. 2007). Briefly, *A. hydrophila* isolates were cultured in TSB supplemented with different concentrations of garlic extracts (1, 2, 4, 6 and 8 mg/mL). Extracts were prepared using distilled water and methanol at the concentrations of 10%, 20% and 40% (v/v). The use of different methanol concentrations was intended to evaluate the effect of solvent polarity on the extraction efficiency. By employing different solvents, the study aimed to identify the most effective solvent for maximising the recovery of antibacterial compounds from the garlic. Tubes containing only aqueous garlic extract served as controls. All inoculated tubes were incubated at 30°C for 24–48 h, after which bacterial growth was assessed by means of visual inspection of turbidity. The lowest concentration of garlic extract that inhibited visible growth was recorded as the MIC.

To determine the minimum bactericidal concentration (MBC), 0.1 mL aliquots were aseptically withdrawn from broth tubes with and without visible growth following incubation in the MIC assay. The samples were subsequently spread onto *Aeromonas* selective agar plates and incubated at 30°C for 24–48 h. The purpose of this subculture step was to verify the results of the MIC assay under solid culture conditions and to determine the lowest concentration of garlic extract that prevented colony formation, which was recorded as the MBC.

### 2.7 Statistical Analysis

All generated data and results from laboratory investigations were entered, cleaned and coded before statistical analysis was conducted. One‐way analysis of variance (ANOVA) was performed, followed by Tukey's HSD post hoc test for multiple comparisons. Statistical significance was set at 5% level (*p* < 0.05). All analyses were carried out in Python (3.13.3) with the pandas and statsmodels libraries.

## 3 Results

### 3.1 Molecular Detection of *A. hydrophila* by PCR

**FIGURE 3:**
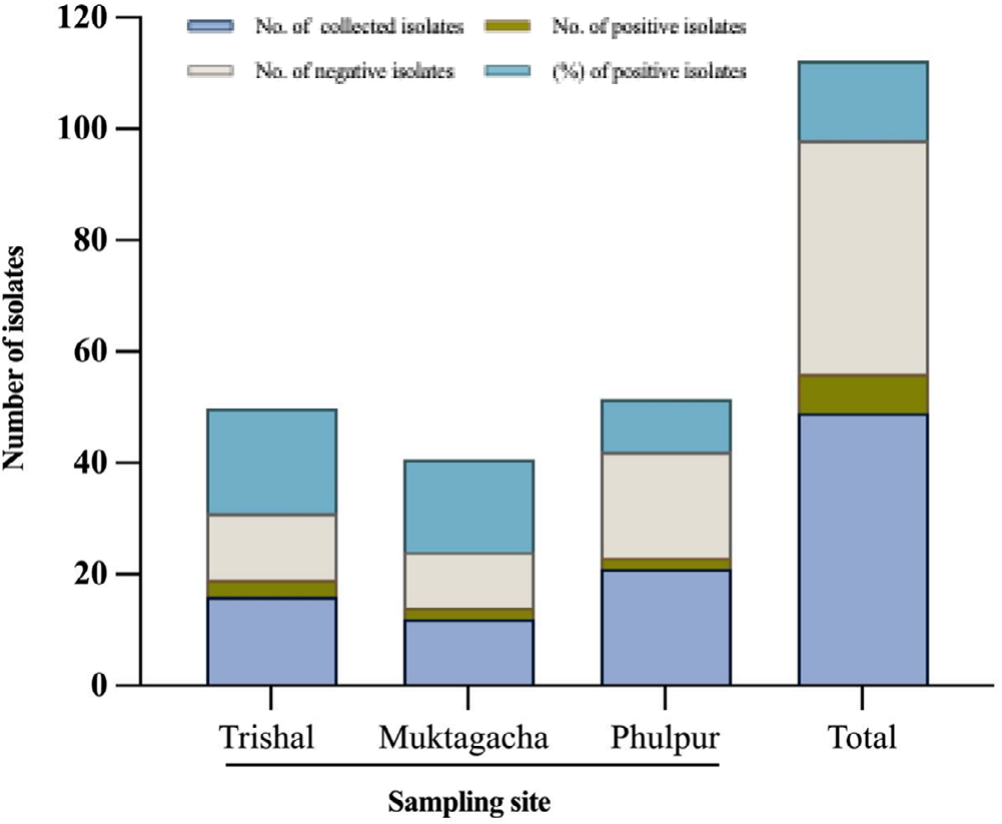
Stacked bar chart illustrating the proportion of *Aeromonas hydrophila*‐positive isolates relative to the total number of isolates collected from each sampling site.

**FIGURE 4:**
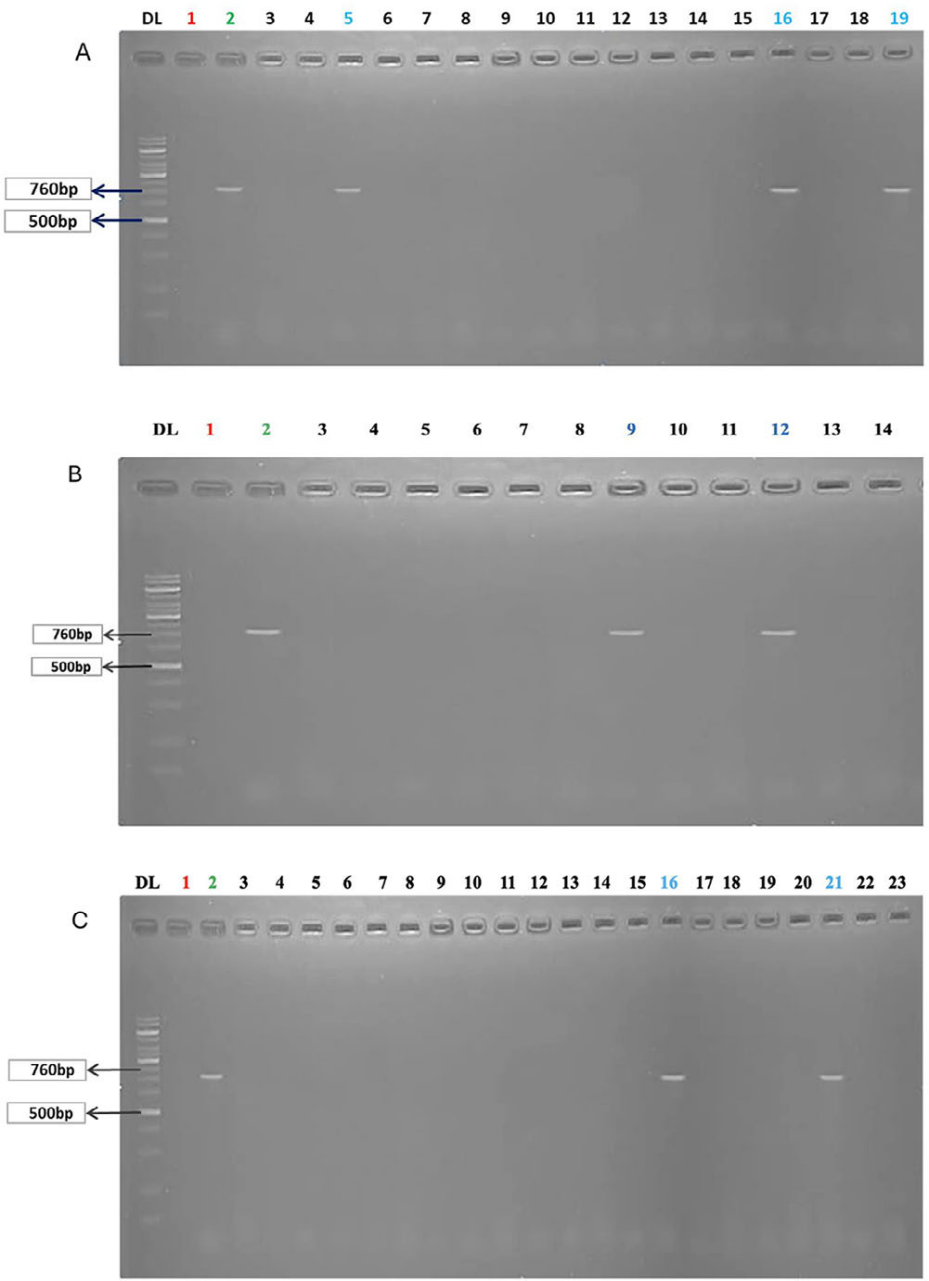
Agarose gel electrophoresis of PCR amplicons targeting the lip gene of *Aeromonas hydrophila* 760 bp, DL: 100 bp DNA ladder; Lane 1: negative control (no DNA); Lane 2: positive control. The other lanes represent positive/negative isolates from (A) Sampling Site 1 (Trishal), Lanes 5, 16 and 19: positive isolates; (B) Sampling Site 2 (Muktagacha), Lanes 9 and 12: positive isolates; and (C) Sampling Site 3 (Phulpur); Lanes 16 and 21: positive isolates.

For the molecular detection of *A. hydrophila*, a total of 49 isolates were collected from three sampling sites: Trishal (*n* = 16), Muktagacha (*n *= 12) and Phulpur (*n *= 21). After the extraction of the gDNA, all the isolates were subjected to PCR with primers targeting the lip gene (760 bp). Following PCR, amplicons were resolved by agarose gel electrophoresis. A visible band of approximately 760 bp was considered positive for *A. hydrophila*. In total, 7 isolates (14.28%) were confirmed as *A. hydrophila* positive. The proportion of positive isolates varied among the sampling sites, with the highest detection in Trishal, where 3 out of 16 isolates (18.85%) were positive, followed by Muktagacha, with 2 out of 12 isolates (16.67%), and the lowest in Phulpur, with 2 out of 21 isolates (9.52%) (Figure 3). Representative gel electrophoresis images from each sampling site are presented in Figure 4.

### 3.2 Inhibitory Effect of Garlic Extract

**FIGURE 5:**
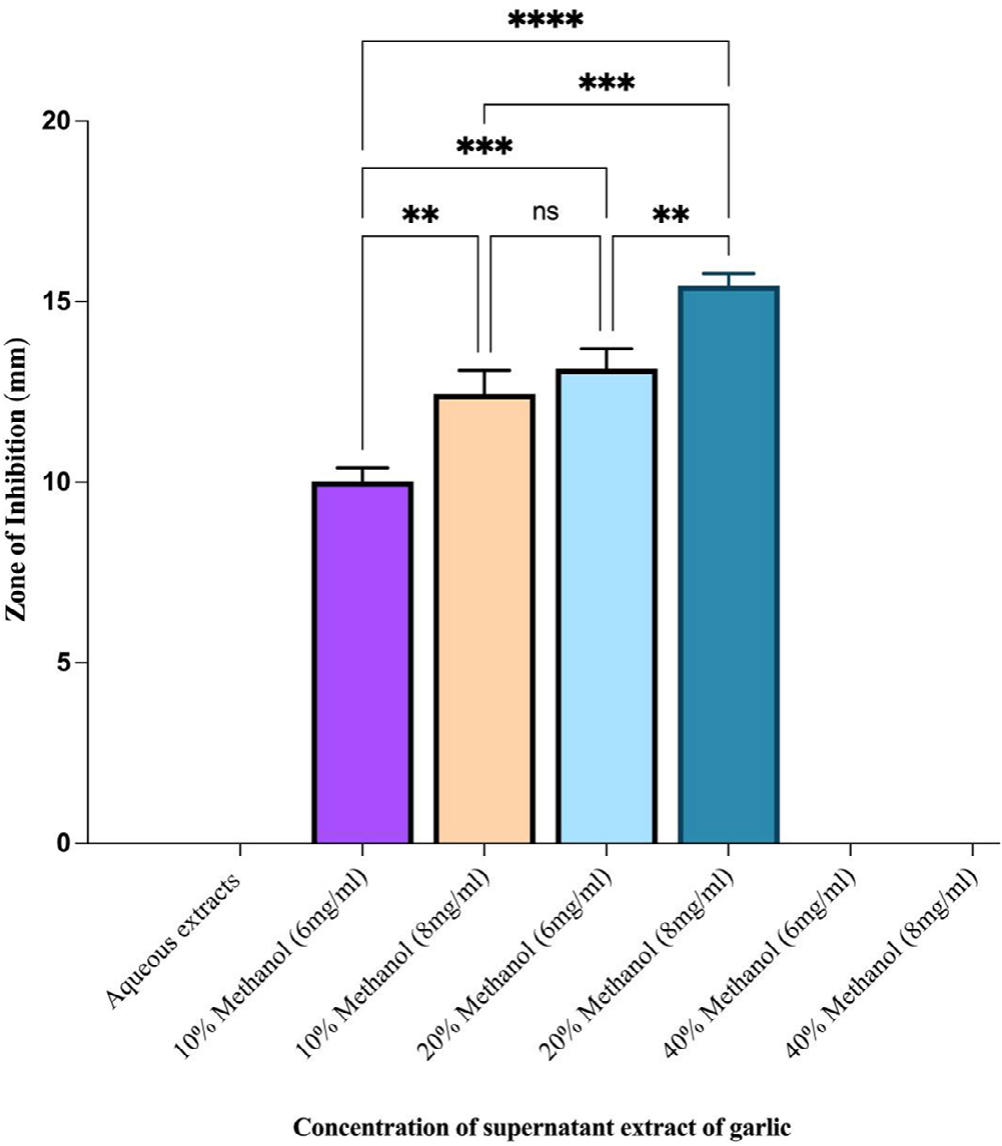
Antibacterial activity of different concentrations of garlic extracts assessed by measuring zones of inhibition (mm). Data are expressed as means ± SEM, and asterisks indicate statistically significant differences determined by one‐way ANOVA.

The antibacterial activity of garlic extracts was strongly affected by both extract concentration and solvent composition. At 6 mg/mL, the garlic extract prepared with 10% methanol produced an inhibition zone of 10.09 ± 0.04 mm, which was notably smaller than that obtained with 20% methanol at the same concentration (13.54 ± 0.04 mm; *p* < 0.001). At 8 mg/mL, the inhibition zone for the 10% methanol extract was 12.67 ± 0.06 mm, which was not significantly different from that of the 20% methanol extract at 6 mg/mL (13.54 ± 0.04 mm; ns) but was significantly smaller than that of the 20% methanol extract at 8 mg/mL (15.11 ± 0.05 mm; *p* < 0.001). The 20% methanol extract at 8 mg/mL showed the highest antibacterial activity, generating a zone significantly larger than that of 6 mg/mL (*p* < 0.0001) and 8 mg/mL (*p* < 0.001) of the 10% methanol extracts, as well as the 6 mg/mL (*p* < 0.01) of the 20% methanol extracts. Interestingly, the 40% methanol extract did not show any antibacterial activity, as no inhibition zones were observed at any tested concentration, which is consistent with the results of the controls (Figure 5).

### 3.3 Minimum Inhibitory Concentration

**FIGURE 6:**
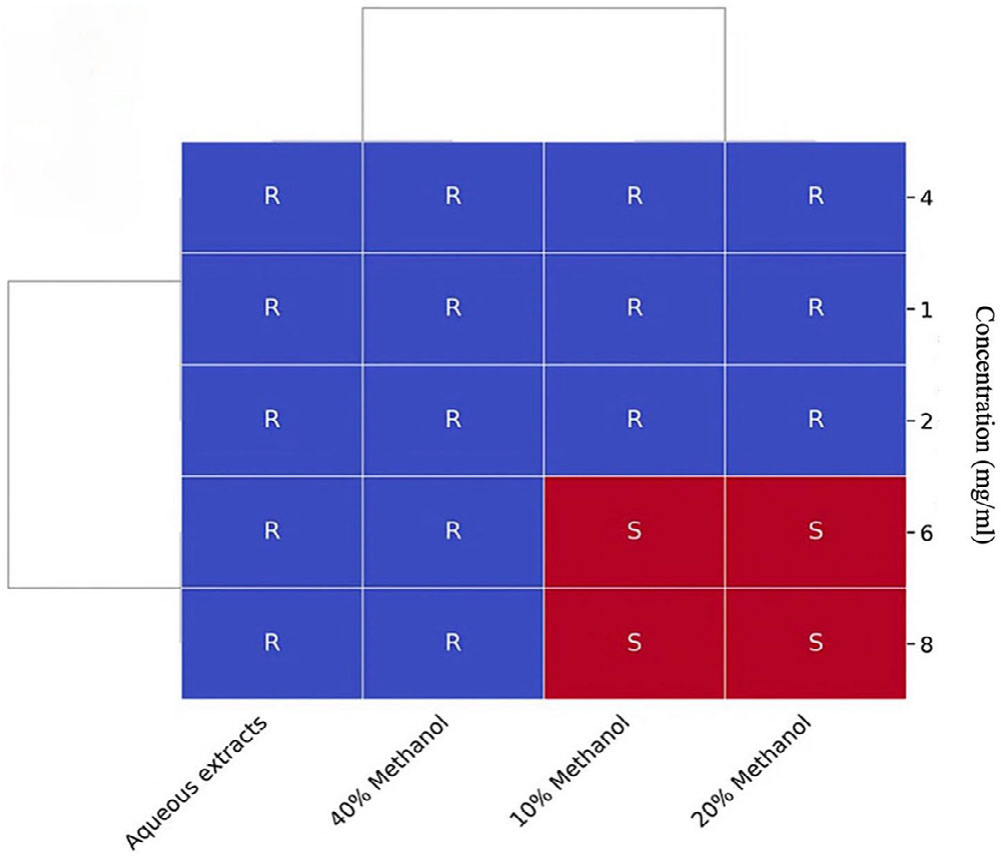
Heatmap with hierarchical cluster analysis showing the minimum inhibitory concentration (MIC) of garlic extracts against *Aeromonas hydrophila* at various concentrations (mg/mL). The blue squares (R) represent resistance (no inhibition), whereas the red squares (S) indicate sensitivity, corresponding to bacterial growth inhibition.

MIC of garlic extract against *A. hydrophila* was determined via the use of both aqueous and methanolic extracts at different concentrations. The aqueous and 40% methanolic extracts exhibited no inhibitory activity at any of the tested concentrations (1, 2, 4, 6, and 8 mg/mL), with all the results classified as resistant (R). In contrast, the 10% and 20% methanolic extracts inhibited bacterial growth at relatively high concentrations and displayed clear antibacterial activity, which was classified as sensitive (S), whereas no inhibitory effect was observed at concentrations below 6 mg/mL (Figure 6).

### 3.4 Minimum Bactericidal Concentration

**FIGURE 7:**
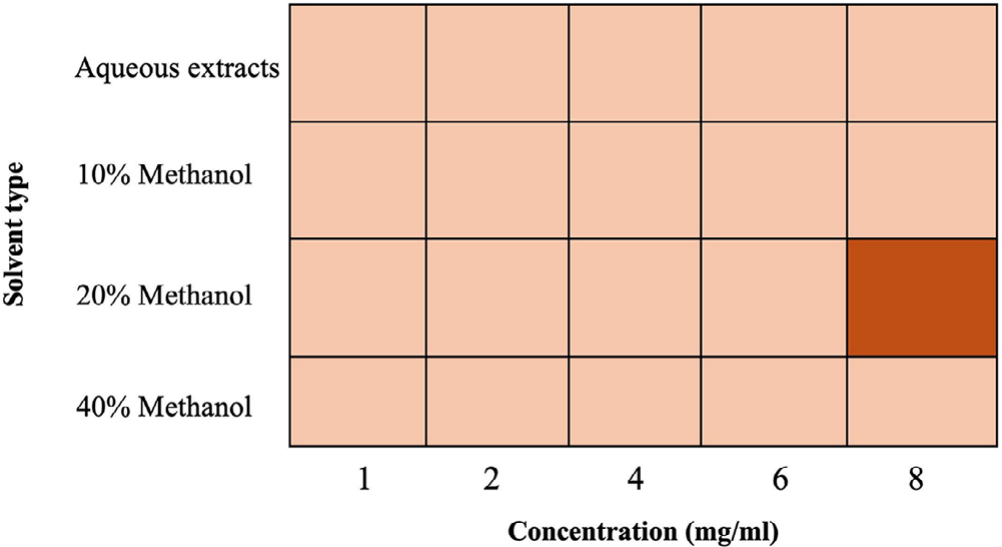
Heatmap showing the *minimum bactericidal concentration* of garlic extracts across different concentrations prepared with various solvents. Colour intensity reflects the bactericidal activity. The *light peach* indicates no bactericidal effect, and the *dark orange* represents bactericidal activity.

MBC of garlic extracts against *A. hydrophila* was also evaluated using aqueous and methanolic extracts at different concentrations. Among the tested extracts, the 20% methanol extract at 8 mg/mL exhibited bactericidal activity with complete inhibition of bacterial growth. In contrast, the aqueous, 10% and 40% methanol extracts had no bactericidal effects at any of the concentrations tested (Figure 7).

## 4 Discussion

*Aeromonas* spp. are widely distributed in freshwater, estuarine and marine environments, where *A. hydrophila* is one of the most frequently isolated species from both diseased and clinically healthy fish (Dias et al. 2016). Rapid and accurate detection of fish pathogens is essential for managing outbreaks and maintaining fish health in aquaculture. In this study, moribund Pangasius catfish infected with *A. hydrophila* exhibited external and internal lesions, including extensive haemorrhages, fin and tail erosion, corneal opacity and body depigmentation, along with other clinical signs consistent with the findings of Ayoub et al. (2021), Al‐Mokaddem et al. (2022), El‐Bahar et al. (2019) and Omar et al. (2016).

Traditional culture‐based pathogen detection methods remain useful, but they are often slow, labour‐intensive and sometimes unable to differentiate closely related bacterial species. Molecular techniques, particularly PCR, have therefore become valuable diagnostic tools due to their higher sensitivity and specificity. For *A. hydrophila*, several genetic markers have been evaluated; among these, the *lip* gene, which encodes a thermostable extracellular lipase, is considered highly specific. Its amplification produces a 760 bp fragment unique to *A. hydrophila* (Swaminathan et al. 2004). In this study, *lip*‐specific primers generated the expected amplicon from *A. hydrophila* genomic DNA, with no amplification observed in non‐target isolates. These results confirm the utility of *lip*‐based PCR as a rapid and species‐specific diagnostic tool that can strengthen epidemiological surveillance in aquaculture.

Detection rates of *A. hydrophila* often vary with sampling strategies, diagnostic methods and fish health status (Khajanchi et al. 2010). In this study, a higher prevalence was observed in Trishal compared to Phulpur and Muktagacha (Figure 3), suggesting possible site‐specific influences such as environmental factors or farm management practices.

This study also evaluated the antimicrobial activity of garlic extracts prepared with different solvents and concentrations. The methanolic extract exhibited stronger antibacterial activity than the aqueous extract (Figure 5), consistent with earlier studies (Vaou et al. 2021; Sheikhlar et al. 2017; Fujisawa et al. 2008). Alcoholic extracts preserve higher levels of sulphur‐containing organosulfur compounds than water‐based extracts, enhancing antimicrobial efficacy. A dose‐dependent effect was observed, with increasing concentrations of methanolic extract producing progressively larger inhibition zones. This effect likely reflects the hydrophobic nature of organosulfur compounds such as S‐allyl cysteine (SAC), S‐allylmercapto‐L‐cysteine (SAMC) and S‐methyl cysteine (Dasari et al. 2022; Rattanachaikunsopon et al. 2007). These stable, non‐volatile compounds retain antibacterial activity, further stabilised by hydroxyl groups present in alcoholic solvents (Amagase et al. 2001).

At 6 mg/mL, inhibition zones were minimal, but 20% methanolic extracts produced slightly larger zones than 10% extracts (Figure 5). The largest inhibition zone (15.11 ± 0.05 mm) was observed at 8 mg/mL in 20% methanol. These results highlight the solvent and concentration‐dependent antibacterial potential of garlic extracts. Aqueous and 40% methanolic extracts showed no activity, likely due to poor solubility, destabilisation or chemical modification of bioactive compounds during preparation (Nakamoto et al. 2020; Dias et al. 2016).

MIC assay indicated that 10% and 20% methanolic extracts inhibited growth only between 6 and 8 mg/mL, while lower concentrations (1–4 mg/mL) were ineffective (Figure 6). The lack of inhibition at lower doses is probably due to the chemical instability and rapid degradation of allicin, the principal bioactive organosulfur compound in garlic (Amagase et al. 2006; Shinkafi et al. 2013). Allicin is highly reactive and rapidly breaks down into secondary sulphur compounds, many of which possess reduced antimicrobial activity (Amagase et al. 2006). Therefore, subinhibitory concentrations may not contain enough allicin to inhibit bacterial growth effectively.

Interestingly, despite using relatively high concentrations of methanolic garlic extract (40%), no inhibitory effect was observed against *A. hydrophila*. This observation is likely explained either by a lack of linear correlation between solvent concentration and antimicrobial efficacy or increasing solvent polarity beyond a certain point may alter the solubility of organosulfur compounds. Previous studies have reported similar findings, showing that both the type and concentration of solvent significantly influence the yield and stability of allicin (Vuddhakul et al. 2007; Christaki et al. 2020). In addition, higher methanol concentrations might cause degradation or instability of organosulfur compounds during extraction, thereby reducing the availability of active antimicrobial molecules (Fujisawa et al. 2008).

Importantly, the 20% methanolic garlic extract at 8 mg/mL not only inhibited growth but also eliminated *A. hydrophila*, indicating its MBC (Figure 7). This bactericidal effect likely reflects allicin's ability to react with thiol groups of key bacterial enzymes (e.g., DNA gyrase, RNA polymerase), disrupt transcription and replication, and damage membrane‐associated proteins, leading to cell lysis (Bhatwalkar et al. 2021; Reiter et al. 2020).

## 5 Conclusion

Overall, this study demonstrates that garlic extract exhibits significant antimicrobial activity against *A. hydrophila* isolated from diseased Pangasius catfish, suggesting it could serve as a natural and eco‐friendly alternative to conventional antibiotics in aquaculture. Both the concentration of garlic extract and the type of solvent considerably affect its antimicrobial efficacy. Therefore, optimisation of extraction conditions is critical for developing garlic‐derived phytobiotics for sustainable fish health management. Further studies are needed to optimise dosage, evaluate long‐term effects and explore its mode of action.

## Author Contributions

The research was conceptualized and supervised by the M.N.H. and M.M.H. The experimental work was carried out by S.S. and M.M.H. The first draft of the manuscript was prepared by S.S., while M.M.H. performed the data analysis and contributed to refining the manuscript. M.N.H. critically reviewed, revised and provided valuable insights into the manuscript for publication.

## Funding

The research fund was supported by the Bangladesh Agricultural University Research System (BAURES), Bangladesh Agricultural University, Mymensingh, Bangladesh.

## Ethics Statement

The authors have nothing to report.

## Conflicts of Interest

The authors declare no conflicts of interest.

## Data Availability

The data that support the findings of this study are available from the corresponding author upon reasonable request.
